# Ribosomal Protein Mutations Induce Autophagy through S6 Kinase Inhibition of the Insulin Pathway

**DOI:** 10.1371/journal.pgen.1004371

**Published:** 2014-05-29

**Authors:** Harry F. Heijnen, Richard van Wijk, Tamara C. Pereboom, Yvonne J. Goos, Cor W. Seinen, Brigitte A. van Oirschot, Rowie van Dooren, Marc Gastou, Rachel H. Giles, Wouter van Solinge, Taco W. Kuijpers, Hanna T. Gazda, Marc B. Bierings, Lydie Da Costa, Alyson W. MacInnes

**Affiliations:** 1 Cell Microscopy Center, Department of Cell Biology, University Medical Center Utrecht, Utrecht, The Netherlands; 2 Department of Clinical Chemistry and Haematology, University Medical Center Utrecht, Utrecht, The Netherlands; 3 Hubrecht Institute, KNAW and University Medical Center Utrecht, The Netherlands; 4 U1009, Institut Gustave Roussy, Université Paris-Sud, Villejuif, France; 5 Department of Nephrology, University Medical Center Utrecht, Utrecht, The Netherlands; 6 Department of Pediatric Hematology, Immunology and Infectious Diseases, Emma Children's Hospital, Academic Medical Center (AMC), Amsterdam, The Netherlands; 7 Division of Genetics and Genomics, The Manton Center for Orphan Disease Research, Boston Children's Hospital, Boston, Massachusetts, United States of America; 8 Harvard Medical School, Boston, Massachusetts, United States of America; 9 Broad Institute, Cambridge, Massachusetts, United States of America; 10 Department of Pediatric Hematology/Oncology, University Medical Center Utrecht, Utrecht, The Netherlands; 11 AP-HP, Service d'Hématologie Biologique, Hôpital Robert Debré, Paris, France; 12 Université Paris VII-Denis Diderot, Sorbonne Paris Cité, Paris, France; 13 U773, CRB3, Paris, France; Stanford University School of Medicine, United States of America

## Abstract

Mutations affecting the ribosome lead to several diseases known as ribosomopathies, with phenotypes that include growth defects, cytopenia, and bone marrow failure. Diamond-Blackfan anemia (DBA), for example, is a pure red cell aplasia linked to the mutation of ribosomal protein (RP) genes. Here we show the knock-down of the DBA-linked *RPS19* gene induces the cellular self-digestion process of autophagy, a pathway critical for proper hematopoiesis. We also observe an increase of autophagy in cells derived from DBA patients, in CD34^+^ erythrocyte progenitor cells with *RPS19* knock down, in the red blood cells of zebrafish embryos with RP-deficiency, and in cells from patients with Shwachman-Diamond syndrome (SDS). The loss of RPs in all these models results in a marked increase in S6 kinase phosphorylation that we find is triggered by an increase in reactive oxygen species (ROS). We show that this increase in S6 kinase phosphorylation inhibits the insulin pathway and AKT phosphorylation activity through a mechanism reminiscent of insulin resistance. While stimulating RP-deficient cells with insulin reduces autophagy, antioxidant treatment reduces S6 kinase phosphorylation, autophagy, and stabilization of the p53 tumor suppressor. Our data suggest that RP loss promotes the aberrant activation of both S6 kinase and p53 by increasing intracellular ROS levels. The deregulation of these signaling pathways is likely playing a major role in the pathophysiology of ribosomopathies.

## Author Summary

Diseases linked to mutations affecting the ribosome, ribosomopathies, have an exceptionally wide range of phenotypes. However, many ribosomopathies have some features in common including cytopenia and growth defects. Our study aims to clarify the mechanisms behind these common phenotypes. We find that mutations in ribosomal protein genes result in a series of aberrant signaling events that cause cells to start recycling and consuming their own intracellular contents. This basic mechanism of catabolism is activated when cells are starving for nutrients, and also during the tightly regulated process of blood cell maturation. The deregulation of this mechanism provides an explanation as to why blood cells are so acutely affected by mutations in genes that impair the ribosome. Moreover, we find that the signals activating this catabolism are coupled to impairment of the highly conserved insulin-signaling pathway that is essential for growth. Taken together, our in-depth description of the pathways involved as the result of mutations affecting the ribosome increases our understanding about the etiology of these diseases and opens up previously unknown avenues of potential treatment.

## Introduction

Diseases linked to mutations affecting the ribosome include inherited disorders such as Diamond-Blackfan anemia (DBA), Shwachman-Diamond syndrome (SDS), and dyskeratosis congenita (DC) [1]. They may also be acquired as with 5q-myelodysplastic syndrome (5q-MDS) [2]. While the phenotypes of these disorders vary extensively in clinical features and severity, the majority of them share some form of cytopenia. DBA, for example, is a pure red cell aplasia linked predominantly to mutations in ribosomal protein (RP) genes [3]. Patients with DBA experience a block in erythroid progenitor cell division and expansion in the bone marrow leading to the characteristic erythroblastopenia [4], [5]. Growth defects, which are routinely observed in animal models of RP gene haploinsufficiency, are also common clinical features of patients with DBA as well as SDS and very severe forms of DC [6]–[10]. While it has been speculated that the hematopoietic phenotype at least in DBA patients is linked to the activation of the p53 tumor suppressor [11], the mechanistic understanding of the pathophysiology underlying DBA and other diseases linked to mutations affecting the ribosome remains incompletely understood.

Autophagy is the highly conserved cellular process of self-digestion that involves the formation of double-membrane structures termed autophagosomes engulfing cytoplasmic proteins and organelles [12]. These autophagosomes then fuse with lysosomes to become autolysosomes, wherein the proteins and organelles are degraded and then either recycled or exocytosed [13]. Autophagy is commonly observed during times of nutrient depletion or starvation and is up regulated in response to oxidative stress or the presence of deleterious organelles and protein aggregates [14]. Autophagy also plays a critical role in erythrocyte maturation. Conditional knockout of *Atg7*, essential for the formation of the autophagosome membrane, yields hematopoietic stem cell failure, erythrocyte cell death, and severe anemia in mice due to the defective removal of mitochondria by autophagy (mitophagy) [15]. Additionally, the targeted deletion of the BCL-2 family member *Nix* results in defective erythroid maturation through impaired mitophagy during terminal erythroid differentiation and also causes anemia in a murine model [16].

The nutrient-sensitive AKT/target-of-rapamycin (TOR) pathway plays a critical role in controlling cell growth and size by stimulating the transcription of a number of factors required for protein translation including RP genes [17], [18]. TOR-dependent autophagy induced by starvation or rapamycin treatment occurs through decreasing the TOR-dependent phosphorylation of Atg13, an event required for association of Atg13 with Atg1 and subsequent autophagosome formation [19]. One important downstream effector of TOR signaling is S6 kinase, whose substrates include the translation machinery elements RPS6, eukaryotic initiation factor 4B (eIF4B), and eukaryotic elongation factor 2 kinase (eEF2K) [20]–[22]. S6 kinase phosphorylation also promotes the formation of autophagosomes, which in combination with the negative regulation of autophagy by TOR provides a balance to prevent cells from excessive self-digestion during prolonged periods of starvation [23].

One of the many activators of AKT and TOR is a phosphorylation cascade initiated by the stimulation of cells with the extracellular growth factor insulin and signaling through the insulin receptor substrate (IRS1) and PI3-kinase to promote the turnover of PIP_2_ to PIP_3_ [24]. The highly conserved insulin pathway is required for the cellular import of glucose, the most vital carbohydrate for activation of the glycolysis pathway and the generation of ATP. AKT activation is as effective as insulin stimulation in inducing the expression of the glucose transporter 1 (GLUT1), the most highly expressed glucose transporter on glycolysis-dependent erythrocytes, while in other cells such as adipocytes insulin-activated AKT promotes the translocation of GLUT4 to the plasma membrane to increase glucose import [25], [26]. Resistance of the insulin pathway to stimulation by extracellular ligands can occur through the over activation of S6 kinase, that in turn directly phosphorylates IRS1 leading to its degradation [27], [28]. This mechanism of insulin resistance is commonly found in obese individuals with type II diabetes, due in large part to increased caloric intake and constitutive activation of the TOR pathway and S6 kinase. A loss of insulin signaling is also known to induce autophagy [29], [30]. While insulin signaling can repress autophagy through TOR phosphorylation of Atg13, a TOR-independent mechanism of repression has been described involving the AKT-dependent phosphorylation and functional inhibition of beclin-1, the yeast homolog of Atg6, an important initiator of autophagosome formation [31], [32]. In this study we set out to determine if the mechanism inducing autophagy from RP loss is linked to the insulin pathway in a TOR-independent manner.

## Results

### Knock down of *RPS19* induces autophagy

**Figure 1:**
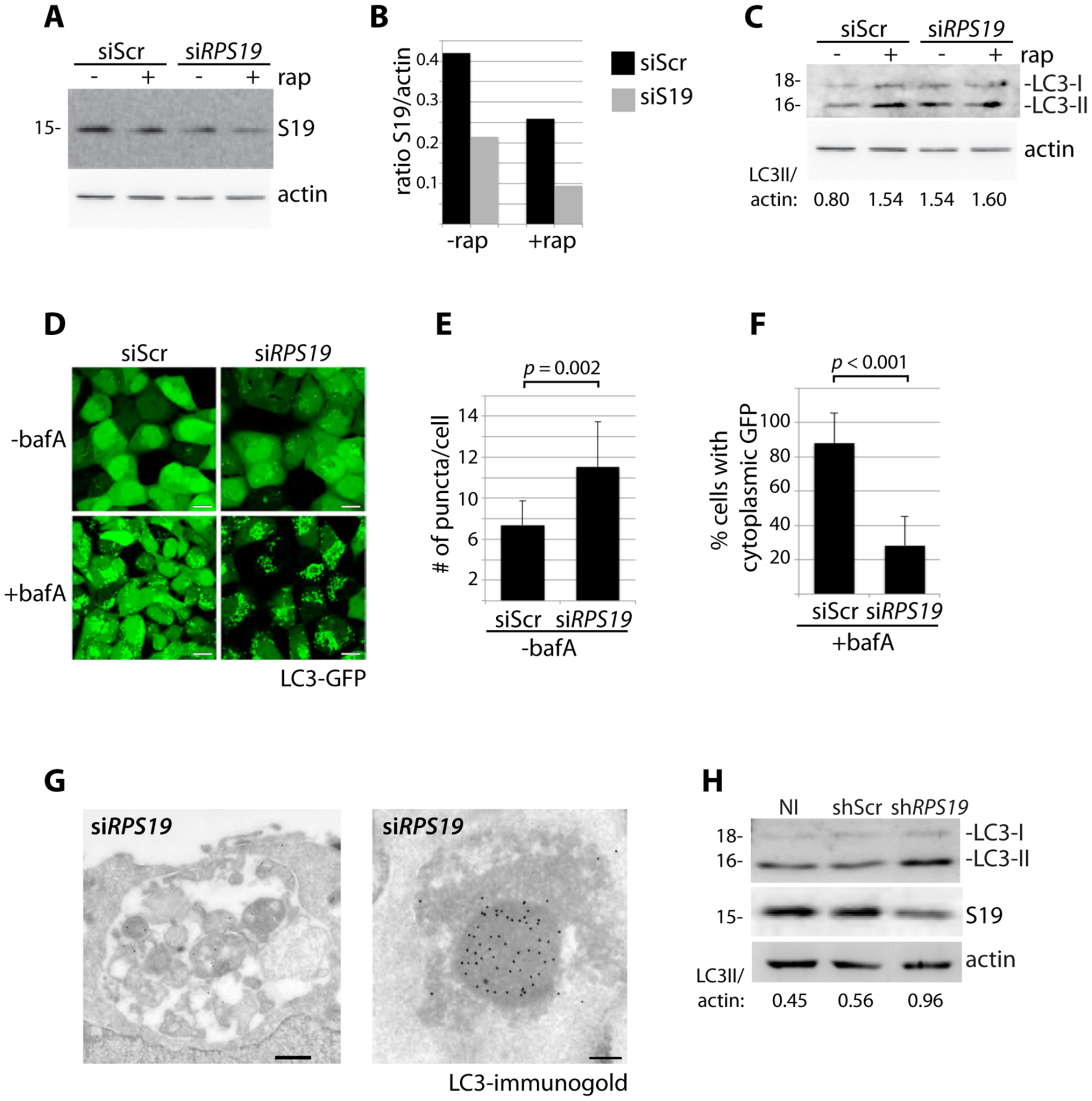
Knock down of *RPS19* induces autophagy. (**A**) Western blot analysis of RPS19 expression in GFP-LC3 HEK cells transfected with siScr or si*RPS19* and either untreated or treated with 100 nM rapamycin overnight (**B**). Densitometer analysis of the ratio of RPS19 to actin expression from Fig. 1A. (**C**) Western blot analysis of LC3 expression in GFP-LC3 HEK cells transfected with siScr or si*RPS19* either untreated or treated with 100 nM rapamycin overnight. (**D**) Confocal analysis of GFP-LC3 HEK cells transfected with siScr or si*RPS19* and either untreated or treated with 50 nM bafilomycin A for 4 hours. Size bars = 10 µM. (**E**) Quantification of the number of GFP-LC3 puncta per cell from Fig. 1D. At least 8 shots from 3 independent transfections are quantified. (**F**) Quantification of the percent of cells in Fig. 1D with cytoplasmic GFP-LC3. (**G**) Representative electron micrographs of GFP-LC3 HEK cells transfected with si*RPS19* and immunogold labeled with LC3 antibodies. Size bar on left panel = 500 nM, on right panel = 200 nM. (**H**) Western blot analysis of LC3 expression in CD34^+^ cells either not infected (NI), infected with a scrambled control (shScr) or infected with a shRNA against *RPS19* (sh*RPS19*).

*RPS19* is the gene most commonly mutated in DBA patients [3]. We therefore selected siRNAs against *RPS19* to study the effects of its knock down in HEK cells stably expressing GFP-LC3, a widely used reporter construct for measuring autophagy levels [33]. Figure 1A shows a western blot analysis of RPS19 expression in GFP-LC3 cells transfected with si*RPS19* or a scrambled siRNA control (siScr) compared to the effects of treatment with 100 nM rapamycin, an inhibitor of TOR that is known to decrease the expression of RPs [18]. The quantification of Figure 1A in 1B indicates that the level of RPS19 knock down obtained is approximately 50%, equivalent to levels expected in DBA patients with *RPS19* haploinsufficiency. Knock down of *RPS19* in GFP-LC3 cells induces autophagy, as shown by western blot analysis with an antibody that recognizes the 18 kD LC3-I and the 16 kD LC3-II protein (Figure 1C). LC3-I is converted to LC3-II upon autophagosome formation due to the conjugation of phosphatidylethanolamine (PE) that enables LC3-II to migrate faster on SDS/PAGE gels, and comparing the levels of LC3-II to actin is a reliable method to measure the level of autophagy [33]. The formation of autophagosomes upon *RPS19* knock down can also be visualized by the increase of GFP-LC3 puncta found in the cytoplasm of si*RPS19* transfected cells compared to cells transfected with siScr (Figure 1D top panels and quantified in Figure 1E as the number of puncta/cell, N>100, *p* = 0.002). Bafilomycin A (bafA) is a drug that inhibits vacuolar H+ ATPase and is commonly used to block the fusion of autophagosomes with autolysosomes [34]. The lower left panel of Figure 1D shows a marked increase in autophagosome formation in GFP-LC3 HEK cells transfected with siScr and treated with bafA as compared to untreated cells, indicating a high degree of steady-state autophagic flux in normal HEK cells. However, a large amount of GFP-LC3 remains cytoplasmic. In cells transfected with si*RPS19* and treated with bafA (Figure 1D, lower right panel) the cytoplasmic GFP-LC3 is almost completely gone and practically all the GFP-LC3 is distributed in a puncta fashion, indicating localization in autophagosomes and/or autolysosomes (quantified in Figure 1F as the percent of cells with cytoplasmic GFP). We confirmed the presence of these structures by immuno-electron microscopy of HEK cells with reduced levels of RPS19 (Figure 1G). These micrographs reveal autolysosomes that contain degraded organelles, including mitochondria (left panel), where we find a strong presence of LC3 using immuno-gold labeling (right panel). Because DBA-linked mutations in *RPS19* primarily affect the function of CD34^+^ erythroid progenitor cells in humans, we infected CD34^+^ cells isolated from cord blood with shRNAs and cultured them in erythroid culture medium. On day nine after isolation, we compared the levels of LC3-II/actin with cells that were not infected (NI), infected with a control shRNA (shScr), or infected with shRNAs against *RPS19* (sh*RPS19*). Figure 1H shows that the successful knock down of *RPS19* is coupled to an increase of the ratio of LC3-II/actin, suggesting an increase in autophagy as a result of RP knock down in CD34^+^ erythroid progenitor cells.

### DBA mutations induce autophagy

**Figure 2:**
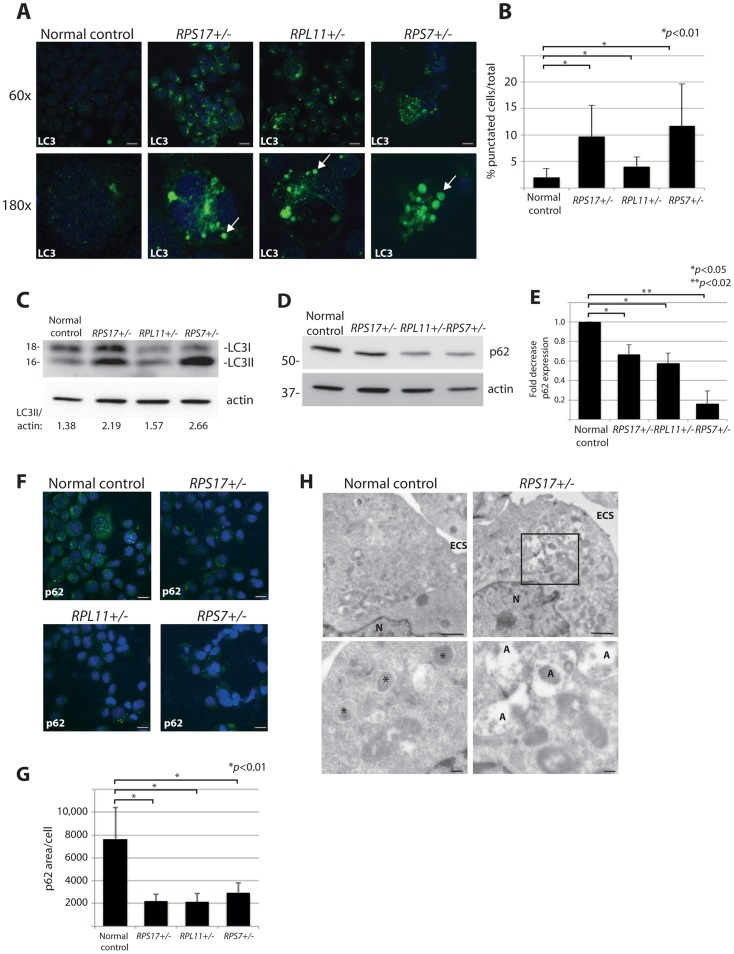
DBA mutations induce autophagy. (**A**) Immunofluorescence with LC3 antibodies in LCLs derived from a normal control or DBA patients. Higher magnifications are represented in the lower panel. Arrows denote puncta indicative of LC3 recruitment to autophagosomes, or accumulation in autolysosomes. Size bars = 10 µM. (**B**) Quantification of the percent of cells revealing LC3 puncta compared to the total number of cells in the 60x shots. (**C**) Western blot analysis of LC3 in DBA LCLs compared to normal controls. The LC3II/actin ratio is determined by densitometer analysis. (**D**) Representative western blot analysis of p62 levels in normal control and DBA patient LCLs. (**E**) Densitometer analysis of p62 protein expression from western blots (N = 3) represented in (**D**). (**F**) Immunofluorescence with p62 antibodies of LCLs derived from a normal control or DBA patients. Size bars = 10 µM. (**G**) ImageJ measurements of p62 expression in (**F**) per total cell area. (**H**) Representative electron micrographs of LCLs derived from a normal control and *RPS17* cells. Control cells have small typically dense lysosomes (*). The much larger autolysosomes (A) are only detected in *RPS17* LCLs. The boxed area in the upper right panel is shown at higher magnification in the lower right panel. N = nucleus, ECS = extracellular space. Bars in top panels = 1 µM, bottom panels = 200 nM.

Another method that allows for visualization of autophagosome formation is immunofluorescence (IF) with antibodies against LC3 [35]. We thus performed confocal IF analysis using lymphoblastoid cell lines (LCLs) derived from DBA patients carrying mutations causing haploinsufficiency of *RPS17*, *RPL11*, or *RPS7*. These IF experiments, illustrated in Figure 2A, reveal distinct puncta representative of LC3-II incorporation into developing autophagosomes and autolysosomes in all three DBA-derived LCLs compared to very few puncta detected in normal cells (quantified in Figure 2B, N>100, *p*<0.01). Additional evidence of increased autophagy in DBA LCLs is provided by western blot analysis revealing an increase in LC3-II/actin ratio in the RP haploinsufficient cells (Figure 2C). The p62 (sequestosome-1/SQSTM1) protein is a major substrate for autophagy that becomes degraded upon increased autophagy [36]. Figure 2D shows a decrease in p62 protein levels by western blot analysis in DBA-derived LCLs compared to normal control cells, 3 independent experiments of which are quantified in Figure 2E. A significant decrease of p62 is also detected by IF in cells derived from DBA patients, which is highly significant when the total amount of p62 expression per cell area is measured (N>100, *p*<0.01) (Figures 2F-G). Lastly, electron micrographs in Figure 2H reveal the presence of many autolysosomes in the *RPS17^+/-^* mutant cells compared to only small lysosomes in the normal control cells. Together, these data indicate that mutation of the RP genes in cell lines derived from DBA patients have increased levels of autophagy.

### Reduction of rpS7 to haploinsufficient levels in zebrafish embryos induces autophagy in RBCs

**Figure 3:**
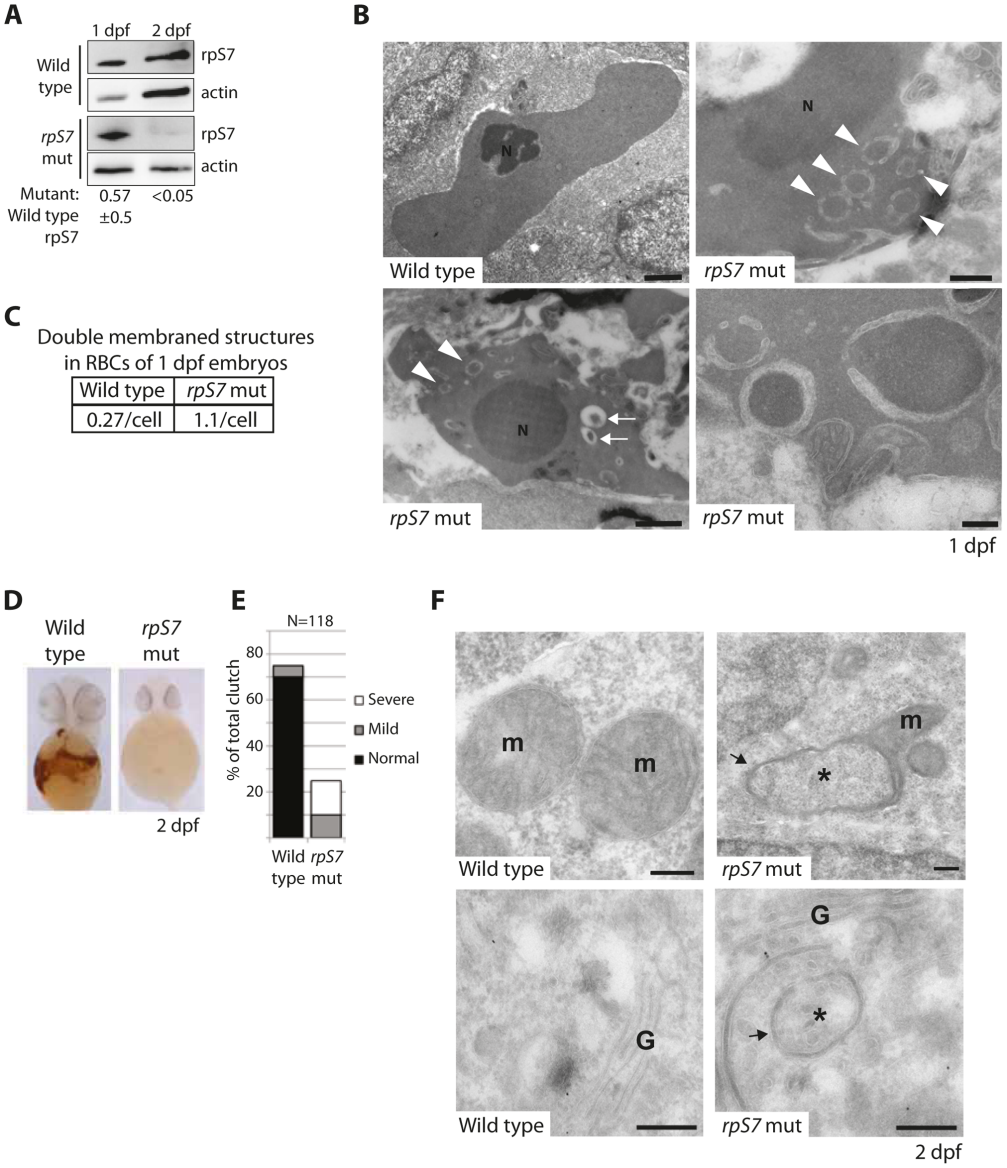
Reduction of rpS7 to haploinsufficient levels in zebrafish embryos induces autophagy in RBCs. (**A**) Western blot analysis of rpS7 protein levels in zebrafish carrying viral inserts in the *rpS7* gene at 1 and 2 dpf. The amount of rpS7 is calculated by densitometer analysis measuring the rpS7/actin ratio (N = 3). (**B**) Electron micrographs of 1 dpf embryos. Representative morphology of RBCs is shown in wild type (upper left panel) and rpS7-deficient embryos (upper right and lower panels). Arrowheads indicate double-membrane autophagosomes, arrows indicate autolysosomes. N = nucleus. Size bars upper and bottom left panels = 1 µM, upper right = 500 nM, lower right = 200 nM. (**C**) Quantification of double-membrane structures in the RBCs of micrographs in (**B**). In each case 28 cells were examined. (**D**) O-dianisidine staining of embryos at 2 dpf. Embryos (N>100) were scored in (**E**) and genotyped to confirm mutation status. (**F**) Representative mitochondrial morphology (m) displayed in wild type (upper left panel) and rpS7*-*deficient embryos at 2dpf (upper right). Typical Golgi complex (G) in wild type embryos shown (lower left panel) compared to Golgi complex in rpS7*-*deficient embryos (lower right). Arrows denote double membranes sequestering cytosolic material, an indication for autophagosome formation. *Denotes engulfed cytoplasmic material. Size bars = 200 nM.

The zebrafish lines we selected to study carry viral inserts in the introns of RP genes that, coupled to the gradual loss of maternally contributed RPs, result in a progressive decrease of RP expression [37]. Although eventually lethal to the embryos, we emphasize that homozygosity of these inserts is synonymous with knock down models of RP gene loss (the only animal models that faithfully recapitulate the anemia phenotype of DBA [38]–[40]) and not deletion mutants (hereafter referred to as RP-deficient embryos). Figure 3A illustrates this using western blot analysis of rpS7 expression in wild type or rpS7-deficient embryos at 1 and 2 days post fertilization (dpf). When normalized to actin, rpS7 expression at 1 dpf in the rpS7-deficient embryos is ∼57% ±5 (N = 3) of the expression level in wild type embryos. By 2 dpf, this drops to less than 5%. We therefore examined the red blood cells (RBCs) at 1 dpf, when rpS7 levels in the mutant embryos are characteristic of *RPS7* haploinsufficiency seen in DBA patients [3].

Figure 3B shows representative electron micrographs of zebrafish RBCs at 1 dpf (which retain their nuclei, unlike human RBCs). The upper left panel is a RBC from a wild type embryo showing mildly condensed chromatin in the nucleus and uniformly distributed, unadulterated cytoplasm. In contrast, the cytoplasm of rpS7-deficient mutant RBCs is mottled with circular double-membrane circular structures, a characteristic feature of autophagosomes (arrowheads in Figure 3B lower left panel, upper right panel, and highlighted in the lower right panel). Additionally, autolysosomes are visible in these mutant RBCs (Figure 3B, small arrows in lower left panel). While small double-membrane structures are occasionally observed in 1 dpf wild type RBCs, they appear much more frequently in the mutant embryos, as quantified in Figure 3C. By 2 dpf the majority of the RBCs in rpS7-deficient embryos have disappeared, evident by staining the embryos for hemoglobin with o-dianisidine in Figure 3D. The results are quantified in Figure 3E by scoring each embryo in the total clutch as either “Normal”, “Mild”, or “Severe” depending on the level hemoglobin loss, followed by genotyping of the individual embryos to confirm true homozygotes. Despite the loss of RBCs in 2 dpf embryos, electron microscopic analysis of rpS7-deficient embryos reveals the presence of characteristic double-membrane structures in other tissues at this stage. These structures originating from both the mitochondria (Figure 3F, upper right panel) and Golgi apparatus (Figure 3F, lower right panel) are shown engulfing cytoplasmic material. Representative images of these organelles in wild type embryos are shown in the left panels of Figure 3F. The mitochondrial structures we visualize in the rpS7-deficient embryos very closely resemble the previously reported mitochondria that supply membranes for autophagosomes in starved mammalian cells [41], and the circular mitochondria that are formed in response to mitochondrial oxidative damage [42].

### RP loss increases phospo-S6 kinase signaling

**Figure 4:**
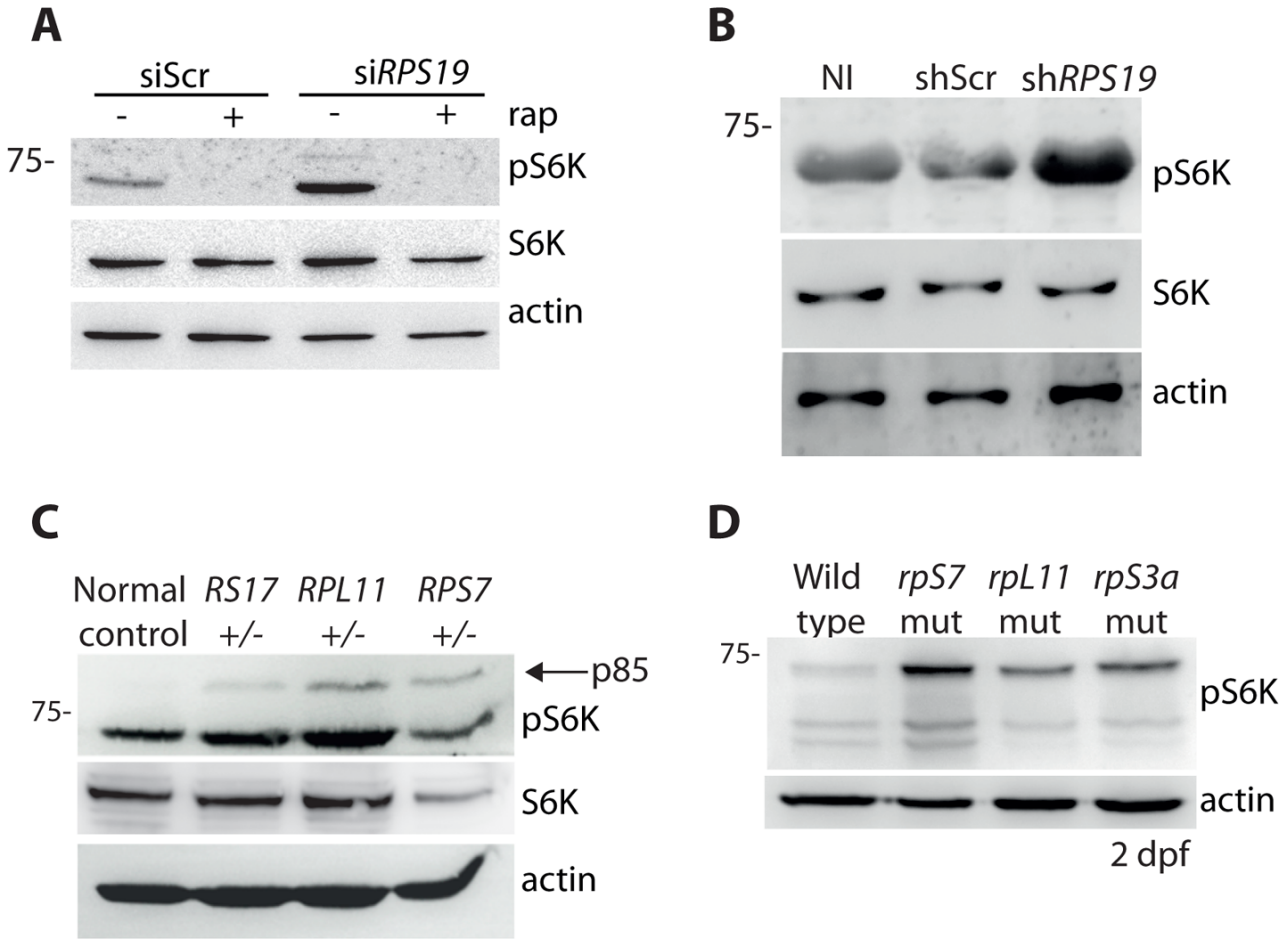
RP loss increases phospo-S6 kinase signaling. (**A**) Western blot analysis showing the expression of phosphorylated S6 kinase in GFP-LC3 HEK cells transfected with siScr or si*RPS19* and either untreated or treated with 100 nM rapamycin overnight. (**B**) Western blot analysis of phosphorylated S6 kinase expression in CD34^+^ cord blood cells infected with a control shRNA (shScr) or an shRNA against *RPS19* (sh*RPS19*). (**C**) Western blot analysis of phosphorylated S6 kinase expression in LCLs derived from a normal control or DBA patients. (**D**) Western blot analysis of phosphorylated S6 kinase expression in 2 dpf zebrafish embryos.

It has been previously reported that the knock down of rpS19 or rpS14 in zebrafish embryos using morpholino oligonucleotides increases phosphorylation of S6 kinase [43]. In agreement with these previous results, we detect an increase in p70 phospho-S6 kinase signaling at Thr389 in the GFP-LC3 HEK cells transfected with the siRNAs against *RPS19* compared to siScr, a signal that is completely abolished by the addition of rapamycin (Figure 4A). Given that rapamycin is well known to directly inhibit TOR, this suggests that TOR is likely phosphorylating S6 kinase in this model. An increase in phosphorylation of S6 kinase is also observed upon knock down of *RPS19* in CD34^+^ cells (Figure 4B). We additionally detect an increase in phospho-S6 kinase signaling in the LCLs with haploinsufficient RP gene mutations, including an increase in p85 S6 kinase phosphorylation (Figure 4C). Similar to human cells with RP loss or mutations, we observe a large increase in S6 kinase phosphorylation in our genetic models of RP deficiency (Figure 4D). Unfortunately, despite several attempts we were unable to find a commercially available S6 kinase antibody that cross-reacted with zebrafish. However, given the strength of the S6 kinase phosphorylation signal and the normal expression of actin, we are confident that this increase is not due to a large increase in S6 kinase expression. Taken together, these results demonstrate that the loss or mutation of several RPs in both human and zebrafish cells results in an increase of S6 kinase phosphorylation.

### RP loss results in a decrease of IRS1 and phosphorylated AKT substrates

**Figure 5:**
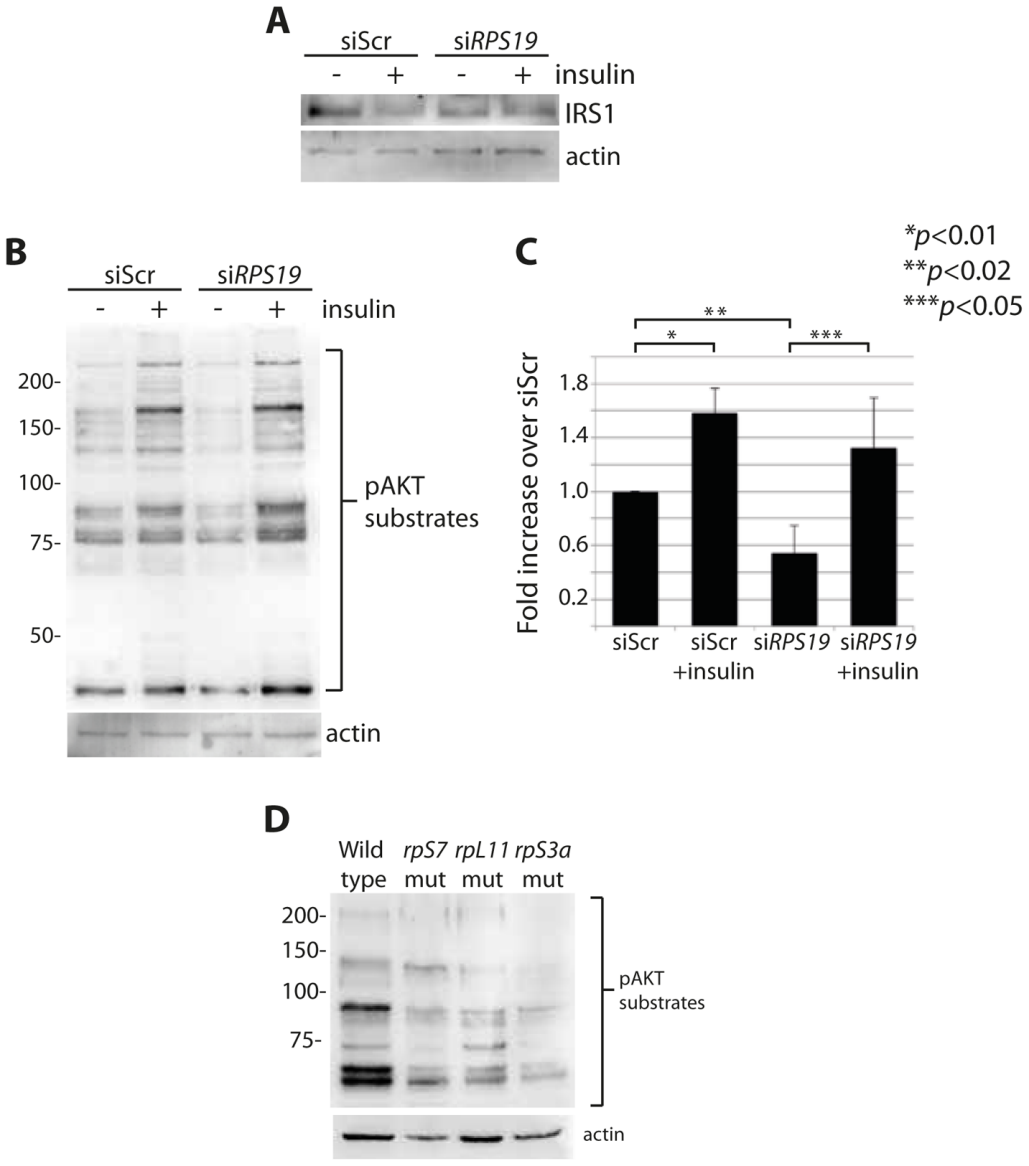
RP loss results in a decrease of IRS1 and phosphorylated AKT substrates. (**A**) Western blot analysis of IRS1 in GFP-LC3 HEK cells transfected with siScr or si*RPS19*. (**B**) Western blot analysis of phosphorylated AKT substrates in GFP-LC3 HEK cells transfected with siScr or si*RPS19* and either untreated or stimulated with 350 nM insulin for 6 hours. (**C**) Densitometer analysis of the total expression level of phosphorylated AKT substrates in (**B**). (**D**) Western blot analysis of phosphorylated AKT substrates in 2 dpf zebrafish embryos.

Overactive S6 kinase phosphorylation causes the inhibition of the insulin pathway, in which AKT plays a central role [27], [28]. One known mechanism of this inhibition is through a direct phosphorylation step of IRS1 by S6 kinase that in turn targets IRS1 for degradation [28]. To determine if RP loss affects downstream mediators of the insulin pathway, we performed western blot analysis on cells with reduced RPs to measure levels of IRS1 and phosphorylated AKT substrates. A decrease of IRS1 is observed in siScr transfected GFP-LC3 HEK cells when stimulated with insulin for 6 hours, similar to what has been shown previously in adipocytes treated with insulin over time [44] (Figure 5A). We also observe by densitometer analysis an ∼50% decrease in IRS1 expression in cells transfected with si*RPS19* compared to siScr, suggesting that the increase in S6 kinase phosphorylation observed in the si*RPS19* cells results in degradation of IRS1. To determine the effect of RP loss on AKT activity, we performed western blotting with an antibody that recognizes the phosphorylation motif of AKT substrates, (RXXS*/T*). Transfecting cells with si*RPS19* resulted in a significant reduction of phosphorylated AKT substrates compared to siScr, while treatment with insulin had the expected effect of increasing the AKT substrate phosphorylation in cells transfected with either siRNA (Figures 5B-C). Similarly, western blot analysis of several different RP-deficient zebrafish embryos reveals a substantial decrease in the phosphorylation of AKT substrates (Figure 5D). Taken together the data suggest that RP loss results in inhibition of AKT phosphorylation activity, likely driven by the degradation of IRS1.

### Insulin abolishes autophagy in cells with RPS19 loss

**Figure 6:**
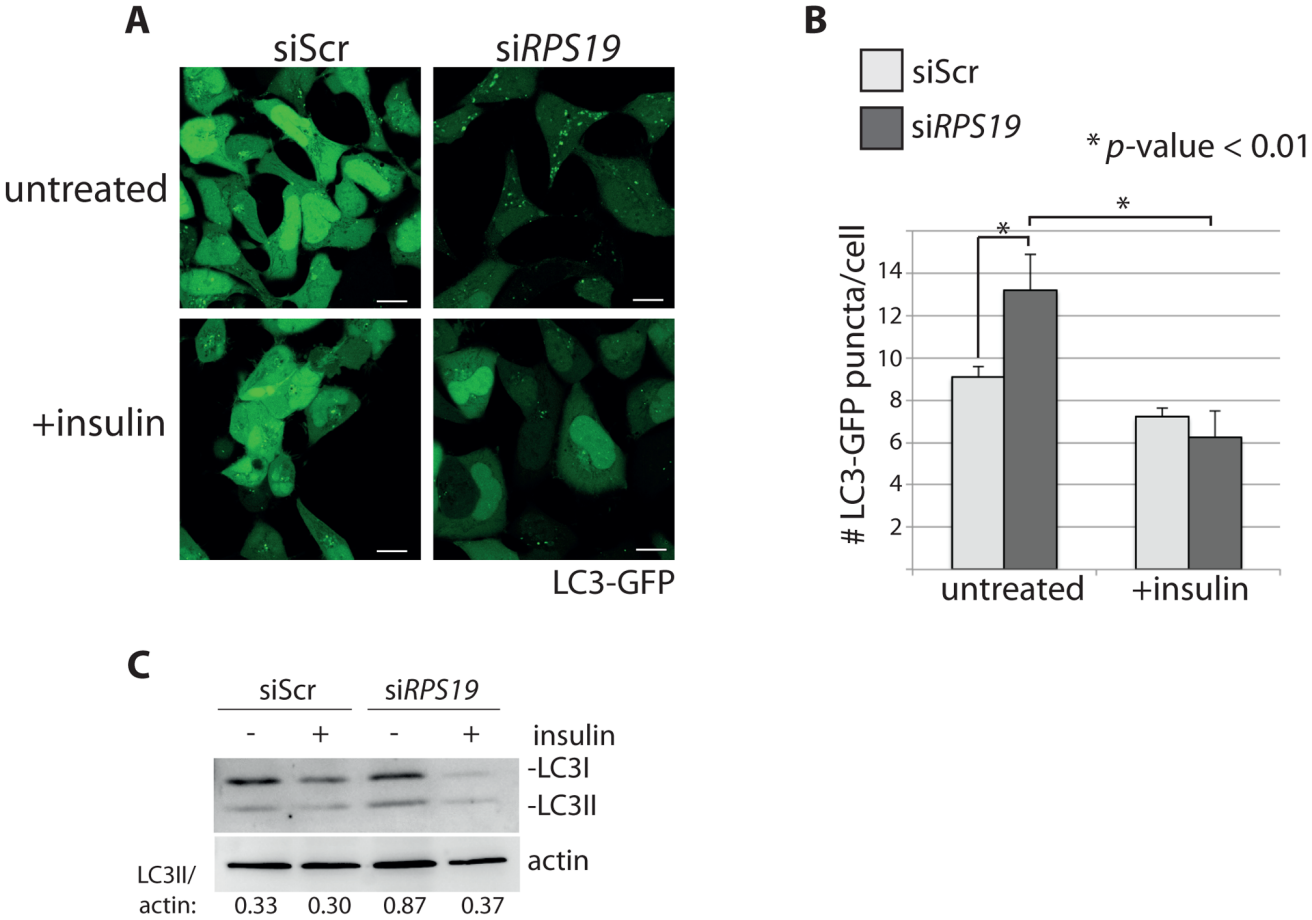
Insulin abolishes autophagy in cells with RPS19 loss. (**A**) Confocal microscopy analysis of GFP-LC3 HEK cells transfected with siScr or si*RPS19* and either untreated or stimulated with 350 nM insulin for 6 hours. Size bars = 10 µM. (**B**) Quantification of the average number of GFP-LC3 puncta per cell in (**A**). (**C**) Western blot analysis of LC3 expression in cells from (**A**). Densitometer analysis used to calculate the ratio of LC3II/actin.

In order to determine if activation of the insulin pathway could override the signals inducing autophagy in models of RP loss, we treated siRNA-transfected GFP-LC3 HEK cells with 350 nM insulin and performed confocal analysis and western blotting. Figures 6A-B show that the addition of insulin significantly reduced the number of GFP-LC3 puncta in the si*RPS19-*transfected cells to the same levels as siScr cells (N>100, *p*<0.01). This reduction of autophagy is also revealed in the LC3 western blots shown in Figure 6C, where the increase of LC3II/actin ratio in the si*RPS19* cells is reduced upon insulin stimulation to the ratio observed in the cells transfected with siScr. When we performed a titration of insulin on LC3-GFP HEK cells transfected with siRNAs against *RPS19* we found that as little as 10 nM insulin was sufficient to significantly reduce the number of autophagosomes compared to untreated cells (Supporting Figure S1). We therefore conclude that the down-regulation of insulin pathway signaling, evident in the data presented in Figure 5, is coupled to the induction of autophagy by RP loss.

### S6 kinase signaling, autophagy, and p53 stabilization are linked to increased reactive oxygen species (ROS)

**Figure 7:**
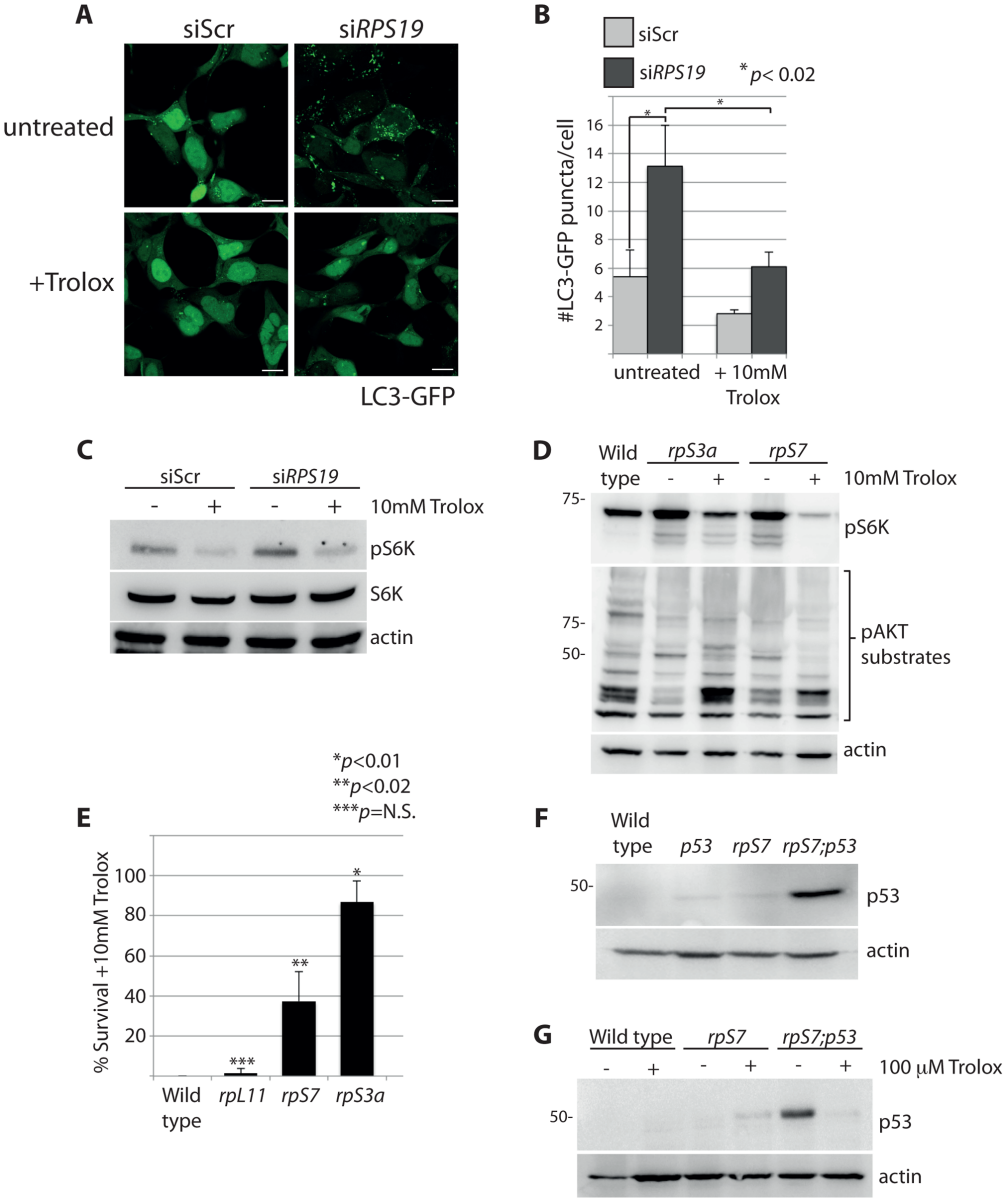
S6 kinase phosphorylation and autophagy is induced by reactive oxygen species (ROS). (**A**) Confocal microscopy analysis of GFP-LC3 HEK cells transfected with siScr or si*RPS19* and either untreated or treated with 10 mM Trolox overnight. Size bars = 10 µM. (**B**) Quantification of the average number of GFP-LC3 puncta per cell in (**A**). (**C**) Western blot analysis of phosphorylated S6 kinase expression in GFP-LC3 HEK transfected with siScr or si*RPS19* and either untreated or treated with 10 mM Trolox overnight. (**D**) Western blot analysis of phosphorylated S6 kinase and phosphorylated AKT substrate expression in 2 dpf zebrafish embryos untreated or treated with 10 mM Trolox overnight. (**E**) Survival rates of embryos treated overnight with 10 mM Trolox. The results are the compilation of three independent experiments with N = 25 embryos representing each mutation. The statistics shown are compared to wild type. (**F**) Analysis of p53 stabilization in 2 dpf wild type, rpS7-deficient embryos, *p53^M214K/M214K^* mutant embryos, or double mutants (*rpS7;p53*) by western blotting. (**G**) Analysis of p53 stabilization by western blotting of zebrafish mutants treated overnight with 100 µM Trolox.

The rapamycin experiment in Figure 4A shows that the increased phosphorylation of S6 kinase linked to RP loss is dependent on TOR. Given the mitochondrial modifications revealed by electron microscopy, we hypothesized that one potential mechanism for this activation may be linked to the TOR function of sensing abnormal levels of intracellular ROS [45]. Trolox, a vitamin E derivative, functions as an anti-oxidant by the intracellular scavenging of ROS [46]. The addition of Trolox to GFP-LC3 HEK cells resulted in a significant reduction in the number of autophagosomes, abolishing the basal level observed in siScr-transfected cells and diminishing the abnormally high level observed in the si*RPS19* cells (N>100, *p*<0.02) (Figures 7A-B). This reduction of autophagy is coupled to a decrease in the phosphorylation of S6 kinase, shown by western blot analysis in Figure 7C. Moreover, when we treated RP-deficient zebrafish embryos with 10 mM Trolox overnight we observed a substantial decrease in the levels of S6 kinase phosphorylation in embryos carrying mutations in *rpS7* or *rpS3a*, which in turn alleviated the inhibition of AKT phosphorylation activity (Figure 7D). Interestingly, the Trolox treatment while clearly blocking autophagosome formation in the GFP-LC3 HEK cells was unexpectedly coupled to an increase of LC3-II by western blotting analysis (Supporting Figure S2), the latter a feature of Trolox that has been previously reported [47]. However, in the same cells we also find that Trolox results in an increase of p62 expression and a decrease of phosphorylated S6 kinase, supporting the confocal results indicating that there is a block of autophagy due to a reduction of S6 kinase signaling (Supporting Figure S2).

The wild type and *rpL11* embryos are not represented in Figure 7D because they do not survive the 10 mM Trolox treatment. This is compared to a survival rate of over 80% of the *rpS3a* embryos and ∼40% of the *rpS7* embryos (Figure 7E). Interestingly, these survival rates correlate with the severity of the morphological phenotypes typically observed with RP mutants including smaller heads/eyes, inflated hindbrain ventricles, and the presence of pericardial edemas [48]. Embryos are morphologically affected most acutely by the *rpS3a* mutation, followed by the *rpS7* mutation, while the *rpL11* mutation is comparatively milder (Supporting Figure S3A). In other words, the increased survival of the *rpS3a* and *rpS7* deficient mutants upon the Trolox treatment is likely due to the their higher initial ROS levels compared to wild type or *rpL11* deficient mutants. This is illustrated in Supporting Figure S3B.

**Figure 8:**
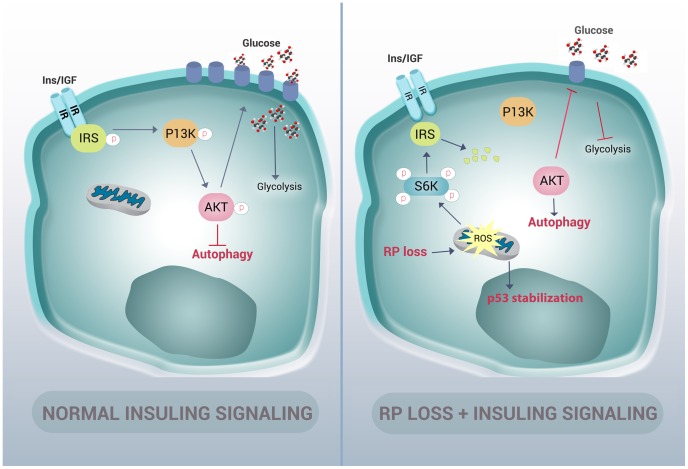
Graphic representation of how RP mutations result in autophagy and insulin pathway inhibition. In normal cells, insulin signals through the insulin receptor substrate (IRS) to the PI3-kinase/AKT pathway in a manner that inhibits autophagy and increases the expression of glucose transporters on the cell membrane. In cells with RP loss, an increase of ROS stabilizes p53 and induces phosphorylation of S6 kinase. This hyper-phosphorylation of S6 kinase results in degradation of IRS and a decrease of insulin signaling to PI3-kinase and AKT. This loss of AKT activation in turn releases the inhibition of autophagy and reduces glycolysis.

Many cellular stresses, including oxidative damage and ROS, result in stabilization of the p53 tumor suppressor and the induction of apoptosis [49]. In zebrafish embryos, a homozygous mutation in the DNA binding domain of the *p53* gene (M214K) allows for an intensified and sustained stabilization of p53 in response to stress [50], [51]. We therefore crossed the rpS7-deficient embryos with the p53 mutant background in order to enhance p53 detection by western blotting (Figure 7F). These double mutant embryos treated with 100 µM of Trolox overnight revealed a considerable reduction of the level of p53 stabilization (Figure 7G). These data suggest that the phosphorylation of S6 kinase, the inhibition of AKT activity, the subsequent induction of autophagy, and the stabilization of p53 due to RP loss are due to an increase of intracellular ROS. This is represented by a graphic illustration in Figure 8.

### Autophagy is not unique to RP gene-linked ribosomopathies

**Figure 9:**
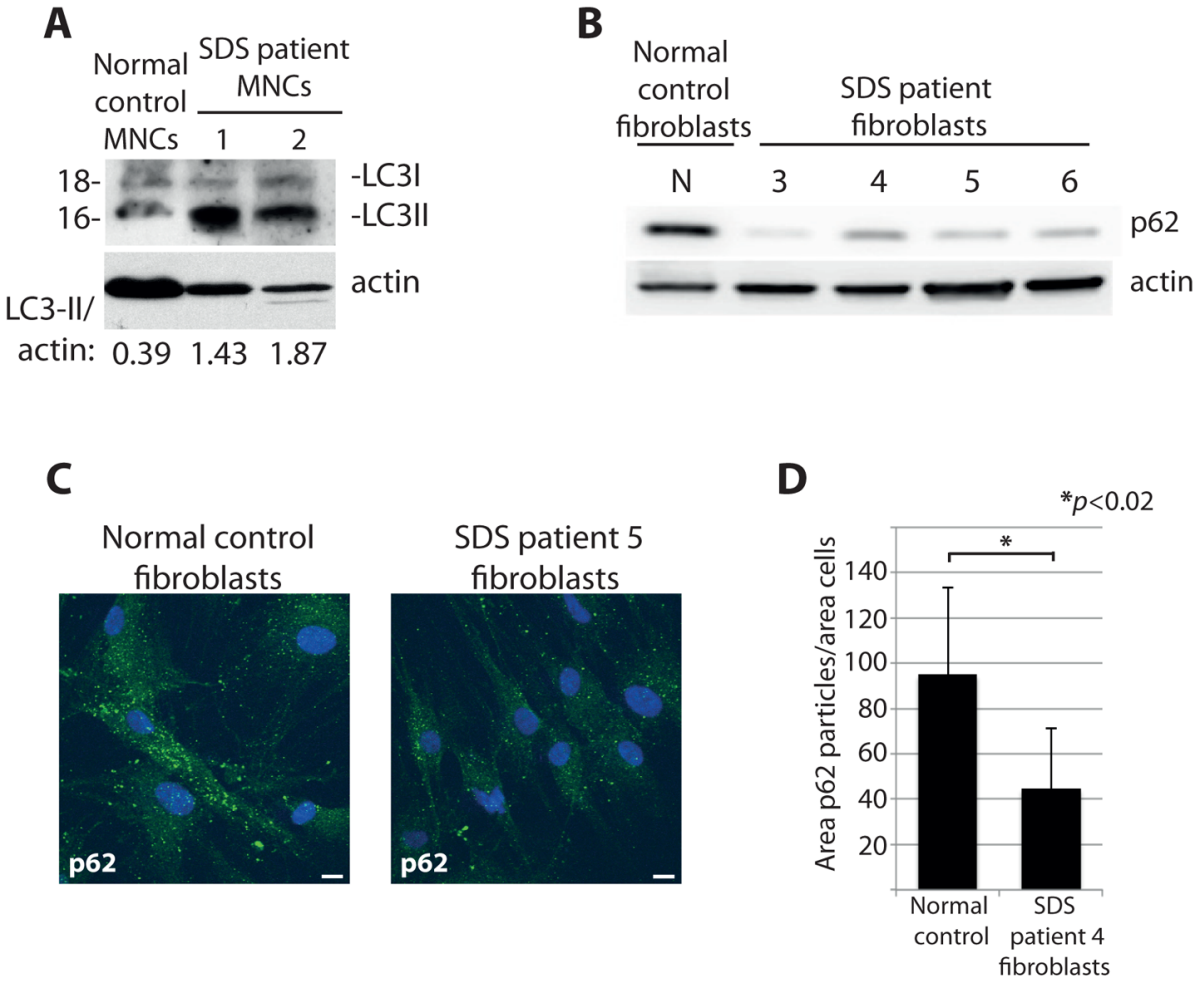
Cells from SDS patients reveal increased autophagy. (**A**) Western blot analysis of LC3 in primary MNCs isolated from two independent SDS patient peripheral blood. (**B**) Western blot analysis of p62 levels in primary fibroblasts isolated from four independent SDS patients. (**C**) Confocal analysis of p62 levels in SDS fibroblasts, quantified in (**D**). At least 8 shots from 3 independent experiments are quantified.

Our study so far focuses on DBA-linked RP gene mutations, however deregulated autophagy may be a more general phenotype of ribosomopathies. To explore this possibility we examined cells derived from SDS patients, most of which carry a mutation in the *SBDS* gene that is important for one of the final maturation steps of the 60S ribosomal subunit [52], [53]. Primary mononuclear cells (MNCs) isolated from the peripheral blood of two independent SDS patients revealed an increase in expression of LC3-II to actin compared to MNCs from a healthy individual (Figure 9A). Moreover, we found evidence of increased autophagy in non-hematopoietic primary fibroblasts derived from four other independent SDS patients. Figures 9B-D illustrate with confocal and western blot analysis that levels of the autophagy substrate p62 are substantially diminished in cultured fibroblasts derived from SDS patients compared to those from a healthy individual. Taken all together, the data suggest that the deregulation of autophagy is a common aspect of ribosomopathies that are linked to bone marrow failure. Moreover, given that the majority of the experiments were performed in non-erythroid cells, they suggest a general relevance of autophagy and inhibition of the insulin pathway that likely extends beyond the cytopenia phenotype of ribosomopathies.

## Discussion

This study demonstrates conclusively that mutations in RP genes induce autophagy through ROS-mediated S6 kinase phosphorylation. Other animal models, including *Drosophila* and zebrafish, have shown previously that different mutations affecting the processing of rRNA induce autophagy, however the mechanisms have remained incompletely understood [54], [55]. The study of the zebrafish defective in rRNA processing by mutation of *pwp2h* suggests that the mechanism is likely p53- and TOR-independent, despite the fact that the authors also note in their mutants an increase in phosphorylation of rpS6 (a substrate of S6 kinase downstream of TOR) [55]. The loss of RPs in zebrafish and mouse models of DBA has also been recently linked to major changes in metabolism, particularly glycolysis [40]. We therefore speculated that a link exists between the autophagy induced by mutations affecting the ribosome and the insulin pathway.

Similar to other zebrafish models of RP loss using morpholinos [56], we observe an increase in phosphorylation of S6 kinase in our genetic RP-deficient zebrafish embryos and in human cells with RP loss which is dependent on TOR and independent of AKT. Since reducing ROS levels with the antioxidant Trolox decreases this S6 kinase phosphorylation and reduces the autophagy activated by RP loss, we propose that increasing ROS levels is the link between RP loss and S6 kinase phosphorylation by TOR. This relationship between ROS, TOR, and S6 kinase has been previously reported as the result of nutrient overload, raising the likelihood that metabolic stress sensed by mitochondria is communicated to the TOR pathway directly through ROS [28], [45].

ROS is increasingly becoming recognized as a critical signaling molecule and not just a damaging agent [57]. Our data underscore this by showing the importance of maintaining ROS levels at a particular threshold during the developmental process, and how reducing this threshold with Trolox is lethal to wild type, but not RP-deficient embryos with severe morphological phenotypes. Interestingly, other recent reports are beginning to reveal links between different inherited bone marrow failure disorders with increasing levels of intracellular ROS including DC, SDS, and Fanconi anemia [58]–[60]. These reports along with our study raise the possibility that the phenotypes of these bone marrow failure disorders may share a common basis that is increased ROS released from stressed mitochondria. Our study goes on to suggest that the stabilization of p53, long regarded as the mechanism behind the early death of erythroid progenitor cells in DBA patient bone marrow, is also triggered by increasing ROS. Since hematopoietic progenitor cell death in Fanconi anemia has been definitively linked to p53 stabilization [61], our results suggest a general mechanism of cell death induced by ROS may underlie these congenital bone marrow failures.

The increase in S6 kinase phosphorylation that we observe upon RP loss is coupled to a decrease in the phosphorylation of AKT substrates, suggesting a mechanism similar to the over activation of S6 kinase promoting insulin resistance. The study mentioned previously is in line with this showing *rpL11^-/-^* zebrafish embryos and whole blood of *rpL11^+/-^* adult fish contain higher levels of glucose compared to wild type controls, an accumulation that would be expected if cells are unable to import and metabolize glucose properly [40]. This same study also measured a decrease of glycolytic enzymes in the *rpL11^-/-^* embryos and in fetal mouse liver cells with knocked down *Rps19* [40]. It has been reported that insulin signaling is important for the proliferation of late erythroblasts and that impaired insulin signaling can cause severe growth defects in animal models [62]–[64]. Therefore insulin pathway inhibition may provide an additional layer of regulation underlying the cytopenia phenotypes, and suggests a putative mechanism of the growth defect phenotypes that are both present in DBA and common to other ribosomopathies.

The conflicting results regarding the Trolox treatment in Supporting Figure S2 are worth noting. As mentioned, the increase of LC3-II that we observe with western blotting analysis despite a clear block of autophagosome formation has been previously reported in cells treated with Trolox, but the results in this report are in the absence of any confocal or IF data showing LC3 localization [47]. Consistent with our results it has been reported that many other antioxidants block autophagy [65]. Therefore it appears antioxidants inhibit autophagosome formation while simultaneously increasing LC3-II accumulation. These data underscore the limitations of using LC3 western blotting as the sole readout of autophagy activity, and highlight the importance of performing concurrent LC3 localization experiments.

Our study describes how the impairment of insulin signaling and deregulation of autophagy result from mutations of RPs. Moreover, we demonstrate that these pathways may be involved in the etiology of a broader range of ribosomopathies that are linked to other mutations affecting the ribosome. While it seems clear that increased levels of ROS as a result of these mutations induces deregulation of autophagy, insulin signaling, and activation of the p53, it is of course difficult to conclusively state the contribution of each impairment to the early death of hematopoietic cells in ribosomopathy patients. Most likely this early death is a combination of all these and other deregulated pathways. Nonetheless, our study suggests that therapeutics targeting intracellular ROS levels may be an effective treatment for patients with bone marrow failures that are linked to mutations affecting the ribosome.

## Materials and Methods

### Ethics statement

Animal experiments were conducted in accordance with the Dutch guidelines for the care and use of laboratory animals, with the approval of the Animal Experimentation Committee (Dier Experimenten Commissie [DEC]) of the Royal Netherlands Academy of Arts and Sciences (Koninklijke Nederlandse Akademie van Wetenschappen [KNAW] (Protocol # 08.2001).

### Fish maintenance

Zebrafish were raised and embryos obtained through natural spawning as previously described (Westerfield, The Zebrafish Book. A Guide for the Laboratory Use of Zebrafish, 3rd Edition, 1995) in accordance with all Dutch regulations and guidelines.

### Cell lines

The GFP-LC3 HEK cells were a kind gift from Dr. Paul Coffer's lab. For the lymphoblastoid cell lines (LCLs), *RPS17* cells carry a deletion in exon 3 (c.200_201delGA) resulting in a frameshift at codon 67 and an early stop codon at 86. *RPL11* cells carry an insert in exon 3 (c.160_161insA) resulting in a frameshift at codon 54 and an early stop at codon 66. *RPS7* cells carry a donor splice site mutation in intron 3 (IVS3+1 g>a). SDS MNCs were obtained by blood samples collected in a heparin vacutainer, diluted 1∶1 in PBS, layered over half the total volume of Ficoll (GE Healthcare) and centrifuged at 287 RCF for 20 min. The MNC and monocyte layer was then removed and washed 2x in 1xPBS. Cell lines were incubated at 37°C +5% CO_2_, the LCLs in RPMI medium +10% FCS +1% pen/strep and fibroblasts in DMEM media +10% FCS +1% pen/strep. All primary cells and patient-derived cell lines used in this study were from patients and families that gave informed consent according to the Declaration of Helsinki.

### siRNA knock downs

siRNAs against *RPS19* were purchased from Invitrogen (#4392420). “Stealth RNAi” scrambled siRNAs from Invitrogen were used as a control (#12935-400). GFP-LC3 HEK cells were plated in 24-well plates (or on glass-bottom confocal dishes, Greiner Bio-One GmbH, #627870) the day before transfection with DMEM media. Transfections were done with Oligofectamine (Invitrogen, #12252-011) according to the manufacturer's instructions using the maximum recommended amount of siRNAs. Bafilomycin A (Sigma, #B1793) was added at a final concentration of 50 nM for 4 hours. Rapamycin (Sigma, #R8781) was added at a final concentration of 100 nM for 6 hours. 10 mM of Trolox (Sigma #238813) was added overnight. Insulin (Sigma, #I6634) was added at a final concentration of 350 nM for 6 hours. Confocal analysis was performed on a Leica SPE microscope. At least 8 shots per transfection were taken, and at least 3 transfections per condition were performed. The counter in ImageJ was used to quantify the number of puncta per cell and the number of cells with cytoplasmic GFP (the images were unlabeled to prevent bias).

### Western blot analysis

Zebrafish embryos were lysed as previously described [66]. Trolox was added to embryos in E3 media overnight at 10 mM. LCLs and fibroblasts were lysed on ice using 1% Triton X-100 buffer containing protease and phosphatase inhibitor tablets (Roche) and normalized to 25 µg per sample. Lysates were loaded and fractionated by SDS-PAGE (8% for IRS1 and pAKT substrates; 10% gels for p62, pS6 kinase, and S6 kinase; and 12% for LC3 and RPS19) under reducing conditions and immunoblotted on PVDF membranes. Primary antibodies used were RPS7 (Santa Cruz #sc-100834) at a dilution of 1∶250, RPS19 (Santa Cruz #sc-100836) at 1∶1000, LC3 for LCLs at 2 µg/ml (Novus Biologicals #NB100-2331), LC3 for MNCs, HEK and CD34+ cells at 1∶1000 (Millipore #ABC232), phospho-S6 kinase (Cell Signaling #92345) at 1∶1000, S6 kinase (Cell Signaling #92025), p62 at 1∶1000 (Progen #GP62-C), and actin antibodies (Santa Cruz #sc-1616) at 1∶200. The zebrafish-specific p53 antibody (zp53) was raised as previously described [66]. Secondary antibodies were diluted 1∶5000 including rabbit-HRP (GE Healthcare) for LC3, pS6 kinase, and S6, mouse-HRP (GE Healthcare) for RPS7, RPS19, and zp53 blots, guinea pig-HRP (Abcam #ab97155) for p62, and goat-HRP (Santa Cruz #sc-2020) for actin blots. ECL reagent (Amersham Biosciences) was used for detection either with Kodak Biomax XAR film or with ImageQuant LAS 4000 (GE Healthcare). Densitometer analysis was performed using a GS-800 densitometer (BioRad) and Quantity One software (BioRad).

### Survival curve

Embryos were left in their chorions and treated with 10 mM Trolox in E3 media overnight. The survival of the embryos was assessed by the presence of a heartbeat.

### Electron microscopy

For electron microscopy analysis, LCLs, GFP-LC3 HEK cells, and 1 and 2 dpf zebrafish embryos were fixed in a mixture of 2% paraformaldehyde and 0.2% glutaraldehyde in 0.1 M phosphate buffer. The cells or embryos were washed in PBS-glycine to quench free aldehydes, then embedded in gelatin and infiltrated in 2.3 M sucrose, followed by rapid freezing in liquid N_2_. 50 nM thick cryosections were cut at −120°C using an Ultracut-S ultra microtome (Leica Microsystems, Vienna, Austria). Sections were either directly viewed in a JEOL 1200CX electron microscope (Jeol Ltd. Japan), or after immuno-gold labeling with rabbit anti-LC3B (Abgent, #AP1805a or AP1806a, Oxfordshire UK), followed by 10 nm protein-A gold.

### Immunofluorescence

For LCLs, Shandon cytospins (5 min at 35 RCF) were used to place cells on 12 mm coverslips (VWR), cells were then fixed and permeabilized with methanol-acetone (1∶1 volume). Fibroblasts were grown overnight in DMEM +10% FCS +1% pen/strep on coverslips, then fixed as above. Primary PBMCs were initially fixed in 2% paraformaldehyde in PBS, then placed on coverslips with Shandon cytospins and permeabilized with 0.1% Triton X-100. Cells were blocked with PBS and 0.2% fish skin gelatin (Sigma) at RT twice for 10 min each. All antibodies were diluted in this blocking buffer. Primary antibodies against LC3B (Abgent, Oxfordshire UK) were diluted 1∶100 and p62 (Progen) at 1∶300, then added to the blocked cells for 30 min at RT. Secondary antibodies GaR-Alexa488 (against LC3B, Invitrogen) and DkaGP-Alexa488 (against p62, Biotium) were all diluted 1∶100 and incubated on the cells in the dark for 30 min at RT. Cells were mounted in Prolong Gold + DAPI (Invitrogen). Subcellular distribution patterns were analyzed using a Zeiss LSM-510 confocal microscope. Individuals counting the number of cells in each confocal slice that displayed the LC3-positive puncta carried out quantifications of the puncta staining. Average numbers were displayed as a percentage of the total number of cells per image (unlabeled images were used to prevent bias). For p62 quantification ImageJ software was used to calculate the total amount of p62 staining per total number of cells using the following parameters: File = 8 bits, threshold = 40–255, size = 0–infinity, and circularity = 0.00–1.00.

### Erythroid cell culture

CD34^+^ cells were isolated from cord blood using the immunomagnetic technique (Miltenyi Biotec, Paris, France). CD34^+^ cells were cultured for two days in presence of 10% FBS, 100 U/ml IL-3, 10 ng/ml IL-6, 25 ng/ml SCF, 10 ng/ml TPO, and 10 ng/ml Flt3-L. A first infection with shRNA-RPS19 compared to non-infected cells or shRNA-Scr infection (all the shRNA generation and lentiviral production is previously described [67]) was then performed at a 50 MOI. For shRNA-RPS19C, a second infection was performed 6 hours after the first infection. Cells were cultured for two more days and GFP-positive cells were sorted using the FACSDIVA Cell Sorter. Sorted cells were switched to the same IMDM medium with SCF (50 ng/ml), IL3 (1 U/ml) and EPO at 1 U/ml till D7 when the FBS concentration was increased to 30% (from D7 to D14). Pellets of 100,000 cells were frozen until lysing.

### O-dianisidine staining

O-dianisidine staining was performed on 2 dpf embryos as previously described [68]. The genotypes of embryos were verified in a 3-primer PCR reaction: 5′-CTCTTGGATGGCTTGGACATGC-3′, 5′- CACTATTTTCGCGCTGGTACTGAAC-3′ (which paired give a 570 bp band with wild type gDNA) and a primer that anneals to the viral insert nLTR3, 5′-CTGTTCCATCTGTTCCTGAC-3′ which yields a 275 bp band in the presence of the viral insertion in the *rpS7* gene.

### Statistics

Statistics in all experiments were performed using the Student's two-tailed t-test.

## Supporting Information

Figure S1 (**A**) Confocal analysis of LC3-GFP expressing HEK cells transfected with siRNAs against *RPS19* and either untreated (upper left) or treated overnight with 10, 50, or 100nM insulin. (**B**) Quantification of the number of LC3-GFP positive puncta per cell. **p<0.01.
(PDF)

Figure S2 Representative western blot analysis of GFP-LC3 HEK cells transfected with siRNAs against *RPS19* or a scrambled control (siScr) and blotted with antibodies against LC3, p62, pS6 kinase (pS6K), S6 kinase, (S6K) or actin. The ratio of LC3-II to actin expression is calculated using densitometer measurements.
(PDF)

Figure S3 (**A**) Representative gross morphology of wild type embryos compared to those homozygous for viral inserts in the *rpS3a*, *rpS7*, or *rpL11* genes. Arrowheads indicate hindbrain ventricle inflation, arrows indicate pericardial edemas. All embryos shown are at 2 dpf. (**B**) Diagram illustrating how the increased ROS levels caused by *rpS3a* and *rpS7* mutations act to protect the embryos against the lethality caused by long-term exposure to the antioxidant Trolox.
(PDF)
