# Supplementary figures and images for: Ribosomal Protein Mutations Induce Autophagy through S6 Kinase Inhibition of the Insulin Pathway
Source: PLoS Genet. 2014 May 29;10(5):e1004371. doi: 10.1371/journal.pgen.1004371 (PMC4038485; doi:10.1371/journal.pgen.1004371)

# Supporting Figure S1

**A**

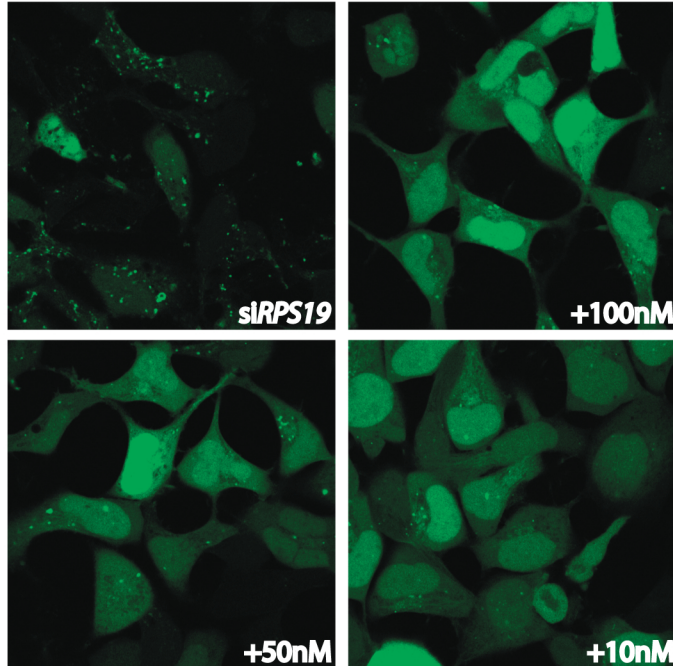

**B**

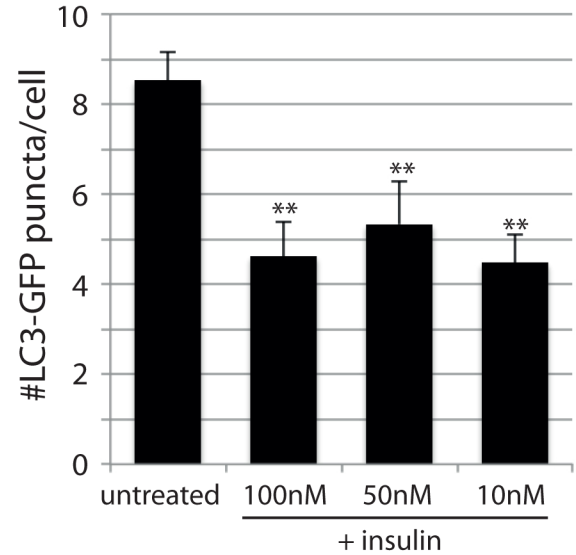

Supplement: Figure S1 — (A) Confocal analysis of LC3-GFP expressing HEK cells transfected with siRNAs against RPS19 and either untreated (upper left) or treated overnight with 10, 50, or 100nM insulin. (B) Quantification of the number of LC3-GFP positive puncta per cell. **p<0.01. (PDF) [file pgen.1004371.s001.pdf]

## Supporting Figure S2

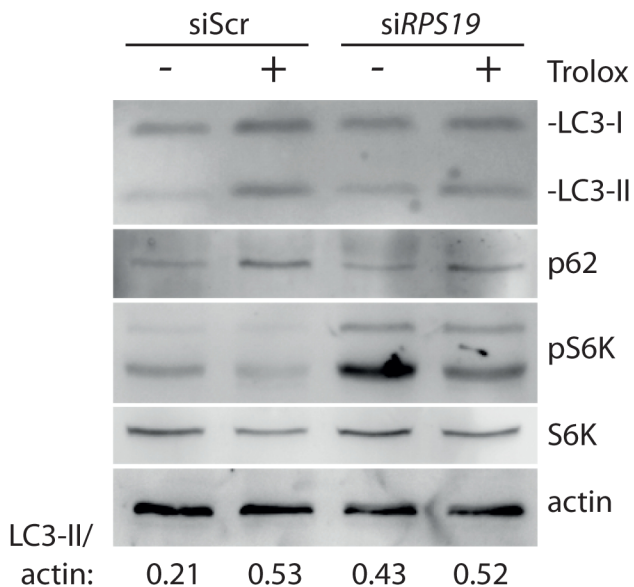

Supplement: Figure S2 — Representative western blot analysis of GFP-LC3 HEK cells transfected with siRNAs against RPS19 or a scrambled control (siScr) and blotted with antibodies against LC3, p62, pS6 kinase (pS6K), S6 kinase, (S6K) or actin. The ratio of LC3-II to actin expression is calculated using densitometer measurements. (PDF) [file pgen.1004371.s002.pdf]

# Supporting Figure S3

**A**

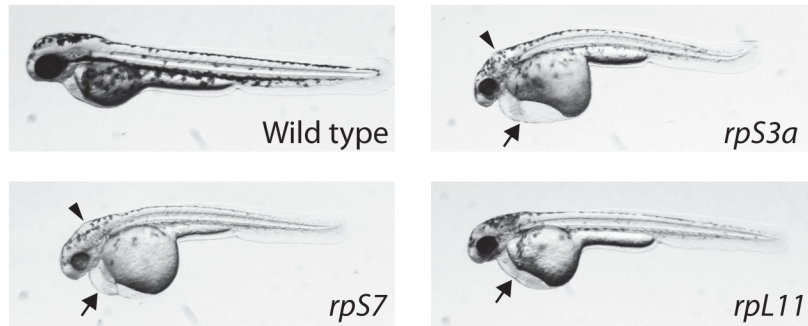

**B**

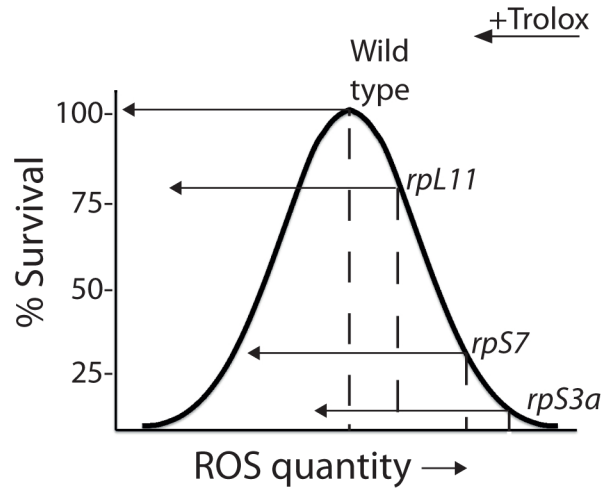

Supplement: Figure S3 — (A) Representative gross morphology of wild type embryos compared to those homozygous for viral inserts in the rpS3a, rpS7, or rpL11 genes. Arrowheads indicate hindbrain ventricle inflation, arrows indicate pericardial edemas. All embryos shown are at 2 dpf. (B) Diagram illustrating how the increased ROS levels caused by rpS3a and rpS7 mutations act to protect the embryos against the lethality caused by long-term exposure to the antioxidant Trolox. (PDF) [file pgen.1004371.s003.pdf]
